# The Regulation of Oxidative Stress Is a Conserved Response to RNA Virus Infection in Fish

**DOI:** 10.3390/antiox15010096

**Published:** 2026-01-12

**Authors:** Alejandro Romero, Patricia Pereiro, Antonio Figueras, Beatriz Novoa

**Affiliations:** Instituto de Investigaciones Marinas (CSIC), Eduardo Cabello 6, 36208 Vigo, Spain; patriciapereiro@iim.csic.es (P.P.);

**Keywords:** reactive oxygen species (ROS), redox balance, antiviral immunity, proteomics, RNA viruses, IPNV, VHSV, RGNNV, fish immunology, aquaculture health

## Abstract

RNA viruses are major pathogens in fish, causing high mortality and substantial economic losses in aquaculture. To uncover conserved antiviral mechanisms, we investigated the response of turbot (*Scophthalmus maximus*) to viral hemorrhagic septicemia virus (VHSV), infectious pancreatic necrosis virus (IPNV), and red-spotted grouper nervous necrosis virus (RGNNV) using a comparative proteomic approach complemented by in vivo and in vitro functional assays. Proteomic analyses revealed the central, conserved role of proteins involved in reactive oxygen species (ROS) production and redox homeostasis during early infection. Functional assays using head kidney-derived leukocytes identified neutrophils and macrophages as the primary ROS producers and showed that the modulation of cytoplasmic and mitochondrial ROS, as well as ROS-dependent DNA release, follows virus-specific patterns. The pharmacological inhibition of NADPH oxidase and mitochondrial ROS significantly affected viral replication, demonstrating the direct role of ROS in viral pathogenicity. Collectively, these findings highlight redox modulation as a conserved host response in teleost fish during RNA virus infection, linking oxidative stress regulation to viral progression. This knowledge provides a foundation for developing broad-spectrum therapeutic or preventive strategies to enhance disease resistance and promote sustainable aquaculture.

## 1. Introduction

Comparative studies analyzing the physiological responses of a single host species to multiple viruses, as well as the responses of multiple host species to the same virus, provide valuable insights into common antiviral mechanisms and open new avenues for the development of broad-spectrum therapeutic strategies [1]. However, such studies are relatively scarce in fish and are mainly based on transcriptomic analyses, without proteomic data or functional validation to confirm whether transcriptional changes translate into protein-level effects [2,3,4,5].

Elucidating the molecular mechanisms underlying shared antiviral responses requires exploring fundamental cellular processes, among which the regulation of redox balance plays a central role in defense against diverse pathogens, including bacteria, parasites, fungi, and viruses [6,7,8,9]. Free radicals, including reactive oxygen species (ROS) and reactive nitrogen species (RNS), are highly reactive, unstable molecules generated during aerobic metabolism [10]. They are mainly produced at the plasma membrane and within cellular organelles such as mitochondria, endoplasmic reticulum, peroxisomes, and lysosomes, through both non-enzymatic reactions (e.g., Fenton and Haber–Weiss) and enzymatic pathways involving the NADPH oxidase (NOX) complex, mitochondrial electron transport chain (ETC), and other oxidoreductases [10,11,12]. Among these, NOX complexes, particularly NOX2 expressed in immune cells such as neutrophils and macrophages, are inducible enzymes that generate ROS upon infection or inflammatory stimuli, transferring electrons from NADPH to molecular oxygen across membranes [13]. In contrast, mitochondria continuously generate ROS during oxidative phosphorylation, mainly at complexes I and III of the ETC, where electron leakage reduces oxygen to superoxide [14]. Although both sources contribute to redox signaling, they are subject to distinct regulatory controls and exert different biological effects.

Moderate to low ROS levels are essential for cellular homeostasis, acting as regulatory molecules in processes such as proliferation, differentiation, apoptosis, metabolism, and immune responses [10]. However, disruption of redox balance leads to oxidative stress, causing irreversible damage to DNA, lipids, and proteins, and promoting inflammation, cell death, and tissue injury. In aquaculture fish, ROS are key for adaptation to environmental changes, pollutants, and husbandry practices, as well as for regulating metabolism, growth, development, and immunity [15,16,17]. Conversely, redox imbalance compromises performance, health, and immune competence [15,18]. To maintain this balance, animals rely on an evolutionarily conserved antioxidant defense system composed of enzymes and small molecules that neutralize free radicals. Among the enzymatic components, superoxide dismutase (SOD) converts superoxide radicals into hydrogen peroxide, catalase (CAT) decomposes hydrogen peroxide into water and oxygen, and glutathione peroxidase (GPx) reduces peroxides using glutathione as substrate [19].

The role of redox balance in host defense is particularly relevant in fish aquaculture, as environmental stressors such as temperature fluctuations, pollutants, and hypoxia can disrupt redox homeostasis, increasing fish susceptibility to infectious diseases and leading to high mortality rates [20]. Viral diseases represent a major threat to sustainable aquaculture due to their high mortality rates, negative effects on growth performance, trade restrictions, and the limited availability of vaccines and antiviral treatments, particularly against RNA viruses [21]. Viruses can profoundly alter cellular redox homeostasis through multiple, interconnected mechanisms [7]. Importantly, ROS production is not universal but depends on the virus type, its replication strategy, and the host cell environment. ROS have been reported to mediate both antiviral [22,23,24] and proviral [25,26,27,28] effects in mammalian and fish viruses. Consequently, the kinetics and magnitude of ROS production are critical in determining whether oxidative processes support viral clearance or, conversely, promote disease progression. Indeed, oxidative stress via RNA virus infections has been reported to impact several aspects of viral disease pathogenesis, including apoptosis, loss of immune function, viral replication, and inflammatory response, among others [29]. In summary, modulation of the oxidative state is a common feature of viral pathogenesis, although its direction and extent are highly virus-specific [7].

Turbot (*Scophthalmus maximus*) is a highly valuable aquaculture species in both Europe and China. Among the most significant viruses for this species are the viral hemorrhagic septicemia virus (VHSV; *Novirhabdovirus*, Rhabdoviridae), the infectious pancreatic necrosis virus (IPNV; *Aquabirnavirus*, Birnaviridae), and the red-spotted grouper nervous necrosis virus (RGNNV; *Betanodavirus*, Nodaviridae) [30,31,32]. The kidney and spleen are major target organs for VHSV, with strong tropism for hematopoietic tissues and endothelial cells. Importantly, VHSV has been shown to infect and replicate in head kidney macrophages and blood leukocytes in several fish species [30]. In the case of IPNV, although the pancreas and liver are primary target organs, the virus disseminates systemically shortly after infection and is detected in circulating leukocytes, reaching its highest replication levels in the head kidney [32]. In contrast, RGNNV exhibits marked neurotropism, primarily targeting the brain, retina, and spinal cord. Although RGNNV can also be detected in non-nervous organs such as the liver, spleen, kidney, and gills, replication in these tissues seems to be limited and may not lead to the production of mature virions [31].

In this context, the objective of this study was to identify conserved mechanisms commonly targeted by RNA viruses using a proteomic approach. Understanding these shared mechanisms provides a foundation for developing broad-spectrum therapeutic or preventive strategies to promote sustainable turbot aquaculture. Our findings reveal that the modulation of the redox balance is a central mechanism consistently affected by all three viruses in this species. We performed both in vitro and in vivo functional assays to evaluate the impact of viral infection on ROS production and to examine how ROS modulation influences viral replication and ROS-dependent neutrophil extracellular trap (NET) formation. This conserved pathway highlights the pivotal role of redox homeostasis in regulating viral replication and orchestrating immune responses.

## 2. Materials and Methods

### 2.1. Animals, Cell Lines, and Viruses

Juvenile turbot (mean lengths: 5.3 ± 0.64 cm and 12.6 ± 1.3 cm) were obtained from Nueva Pescanova (Galicia, Spain). The fish were maintained in 500 L tanks equipped with a recirculating seawater system (35 g/L salinity) at 18 °C under a 12:12 light-dark photoperiod. They were acclimated for two weeks and fed daily with a commercial dry pellet (GEMMA Diamond 1.5, Skretting, Burgos, Spain). A total of 352 fish were used in the study. Tricaine methane sulfonate (MS-222, Sigma Aldrich, Madrid, Spain) was used for anesthesia during injections (50 mg/L) and for euthanasia prior to sampling (500 mg/L). All experimental procedures were approved by the CSIC National Committee on Bioethics (approval code: ES360570202001/21/FUN.01/INM06/BNG01; approval date: 25 November 2021).

The VHSV strain UK-860/94 [33] was propagated in EPC cells (epithelioma papulosum cyprini; ATCC CRL-2872) at 15 °C in Eagle’s minimum essential medium (MEM; Gibco, Thermo Fisher Scientific, Madrid, Spain) supplemented with penicillin (100 IU/mL), streptomycin (100 μg/mL) (P/S; Gibco, Thermo Fisher Scientific, Madrid, Spain), and 2% fetal bovine serum (FBS; Gibco, Thermo Fisher Scientific, Madrid, Spain). The IPNV strain TB-306 [34] was propagated in RTG-2 cells (rainbow trout gonad; ATCC CCL-55) at 20 °C in MEM containing P/S and 2% FBS. The RGNNV strain 475-9/99 [35] was cultured in SSN-1 cells (snakehead fish; ECACC 96082808) at 25 °C in Leibovitz’s L-15 medium (Gibco, Thermo Fisher Scientific, Madrid, Spain) supplemented with P/S, 2% FBS, and L-glutamine (Gibco, Thermo Fisher Scientific, Madrid, Spain).

Viral titers were determined using the Reed and Muench method [36] and expressed as the viral dose that induces cytopathic effect in 50% of cell cultures inoculated with 10-fold serial dilutions of the viral supernatants (TCID_50_/mL).

### 2.2. Turbot Infection and Sampling Procedures

Fish were randomly distributed among tanks, and treatments were randomly assigned to tanks. For the proteomic analysis, a total of five groups of 12 fish (mean length: 5.3 ± 0.64 cm) were inoculated as follows: three groups were intraperitoneally (ip) injected with 50 μL of culture medium (control group) or viral suspension containing VHSV at 1.58 × 10^5^ TCID_50_/mL or IPNV at 1.55 × 10^6^ TCID_50_/mL. The two remaining groups were intramuscularly (im) injected with 50 μL of culture medium (control group) or viral suspension containing RGNNV at 1.58 × 10^5^ TCID_50_/mL. At 3 days post-infection (dpi), head kidneys were collected and samples from four fish were pooled, yielding three biological replicates (four fish per replicate). For RGNNV-infected fish, head kidney and brain samples were obtained from control and infected groups following the same procedure. All samples were sent to BGI (Beijing Genomics Institute, Shenzhen, China) for proteomic analysis.

For the gene expression analysis, samples were obtained as described in [5]. Briefly, another five experimental groups, each consisting of 36 fish, were inoculated as previously described. At 1, 3, and 5 dpi, head kidneys were collected, and tissues from three fish were pooled to obtain four samples. Samples were stored at −80 °C until RNA isolation.

To analyze the in vivo effect of viral infections on ROS-related immune responses, a total of five fish were infected with each of the three viruses. Moreover, two control groups (*N* = 5 each) were intraperitoneally (ip) or intramuscularly (im) injected with culture medium following the same protocols described for the proteomic and transcriptomic analyses. At 3 dpi, total leukocytes were isolated from the head kidney, and ROS production (total and mitochondrial forms) and NET release were evaluated as described in Section 2.10 and Section 2.11, respectively. No animals or experimental data were excluded from the analyses.

### 2.3. Protein Extraction and Quality Control

Samples were weighed and transferred to 2 mL tubes containing extraction buffer (1× protease inhibitor cocktail, SDS L3, EDTA, 10 mM DTT) and two steel beads. Tissues were homogenized in a grinder at 60 Hz for 2 min and centrifuged at 25,000× *g* for 15 min at 4 °C. DTT (10 mM) was added to the supernatants, which were then incubated in a water bath at 56 °C for 1 h. Subsequently, samples were incubated with iodoacetamide (55 mM) in the dark for 45 min. Proteins were precipitated for 30 min at −20 °C with 1:5 cold acetone, and supernatants were discarded after centrifugation at 25,000× *g* for 15 min at 4 °C. Pellets were air-dried and solubilized in extraction buffer without SDS L3. Samples were homogenized, centrifuged again, and the resulting supernatants, containing the protein extracts, were collected.

Protein concentrations were determined using the Bradford method. A standard curve of bovine serum albumin (BSA) ranging from 0 to 0.2 μg/μL was prepared in a 96-well microtiter plate. Samples and standards were stained with Coomassie Brilliant Blue G-250 (Sigma Aldrich, Madrid, Spain), and optical density at 595 nm (OD_595_) was measured using a microplate reader. Protein concentration for each sample was calculated from the standard curve. The quality of the extracted proteins was also evaluated. Ten μg of proteins were mixed with buffer, loaded onto a 12% SDS-polyacrylamide gel, and separated by electrophoresis (80 V for 30 min, followed by 120 V for 120 min). Gels were stained and de-stained using an automated protein staining instrument, and images were scanned.

### 2.4. Proteolysis, High-Performance Liquid Chromatography (HPLC), and Mass Spectrometry Detection

Protein extracts (100 μg) were washed three times as follows: extracts were loaded into 10 kDa ultrafiltration tubes, mixed with 100 μL of 0.5 M TEAB buffer, and centrifuged at 12,000× *g* for 20 min at 20 °C. Proteins were digested with trypsin at a 1:20 enzyme-to-protein ratio by incubating the samples at 37 °C for 4 h. Peptides were collected by centrifugation at 12,000× *g* for 15 min and freeze-dried for further analysis. Freeze-dried peptides were reconstituted in 2% acetonitrile and 0.1% formic acid (mobile phase A). Before separation, peptides were enriched on a trap column and desalted. Peptide separation was performed using a nanoliter liquid chromatograph (Shimadzu LC-20AD) coupled to a tandem self-packed C18 column (75 μm internal diameter, 3 μm particle size, 15 cm column length) at a flow rate of 300 nL/min. The gradient elution for mobile phase B (98% acetonitrile, 0.1% formic acid) was as follows: 0–6 min, 6%; 6–40 min, linear increase to 25%; 40–48 min, increase to 40%; 48–51 min, increase to 90%; 51–55 min, hold at 90%; 55.5–60 min, return to 6%.

Peptides were analyzed using a TripleTOF 5600 mass spectrometer (SCIEX, Framingham, MA, USA) equipped with a Nanospray III ion source and a quartz emitter (New Objectives, Woburn, MA, USA). Data were acquired in high-sensitivity mode under the following conditions: spray voltage, 2300 V; nitrogen pressure, 35 psi; spray gas, 15; interface temperature, 150 °C. The MS1 scan range was 350–1500 Da with a cumulative scan time of 250 ms. The 30 most intense ions (charge states 2–5) were selected for fragmentation, with an MS2 scan range of 350–1250 *m*/*z* and an accumulation time of 50 ms. Dynamic exclusion was set to prevent the same parent ion from being fragmented more than twice within a 12 s window. Collision energy was applied using the rolling collision energy setting.

The mass spectrometry proteomics data have been deposited to the ProteomeXchange Consortium via the PRIDE [37] partner repository with the dataset identifier PXD06803.

### 2.5. Protein Identification and Differential Abundance Analysis

MaxQuant software v.1.5.3.30 [38], integrating the Andromeda search engine, was used for peptide identification and protein quantification. The original MS data were used as input, and the search parameters were set as follows: enzyme, trypsin; peptide mass tolerance, 0.2 Da; fragment mass tolerance, 10 ppm; minimal peptide length, 7; PSM-level FDR, 0.01; protein-level FDR, 0.01; fixed modification, carbamidomethyl (C); variable modifications, oxidation (M), acetylation (protein N-terminus), deamidation (NQ), and pyro-glutamate formation from glutamine (Gln → pyro-Glu). Proteins predicted from different turbot genome sequencing/assembly versions (GenBank accessions GCA_022379125.1, GCA_003186165.1, GCA_013347765.1, GCA_963854745.1) were used for annotation.

Quantitative protein analysis was based on peak intensity, peak area, and LC retention time of peptides from first-order mass spectrometry data. Protein quantification was achieved by aggregating data from peptides assigned to the same protein. For differential expression analysis, comparisons were made between defined groups (VHSV vs. Control_IP, IPNV vs. Control_IP, and RGNNV vs. Control_IM in kidney and brain samples). Fold changes in protein abundance were calculated for each comparison, and significance was assessed using Welch’s *t*-test. Proteins with a fold change > 1.5 and an adjusted *p*-value < 0.05 were considered significantly differentially expressed (DEPs).

### 2.6. Volcano Plots, Venn Diagrams, Heatmaps, STRING Protein–Protein Interaction Networks, and Gene Ontology (GO) Enrichment Analyses

Volcano plots of the DEPs in the comparisons of interest were generated in RStudio v. 4.0.4 using the ggplot2 package [39]. Venn diagrams were constructed with the Venny v.2.1 tool [40]. Heatmaps (average linkage, Euclidean distance) were generated from intensity values using the Clustvis v. 1.0 web tool [41]. Protein–protein interactions were analyzed with STRING v.12.0 [42]. GO enrichment analysis of DEPs was performed in OmicsBox v.1.3.11 platform using Fisher’s exact test with a false discovery rate (FDR) ≤ 0.05.

### 2.7. RNA Isolation, cDNA Synthesis, and qPCR Analyses

RNA was extracted using the SimplyRNA Tissue Kit (Promega Biotech Iberica, Madrid, Spain) in the automated Maxwell RSC 48 Instrument (Promega Biotech Iberica, Madrid, Spain), following the manufacturer’s protocol. RNA concentration was quantified with a Qubit 4 fluorometer (Thermo Fisher Scientific, Madrid, Spain). cDNA was synthesized using the NZY First-Strand cDNA Synthesis Kit (NZYtech, Lisboa, Portugal) from 0.5 μg of total RNA.

Gene expression analysis and viral replication detection were performed by quantitative PCR (qPCR) using primers designed specifically for this study, and their specificity and amplification efficiency were validated. Primer sequences are listed in Supplementary Table S1 and include the following genes: *sod2*, *sod3*, *gpx3*, *ndufs1*, *noxo1b*, *ncf1*, *ncf2*, and *ncf4*. For viral detection, the VHSV glycoprotein (*G*), the IPNV recombinant viral protein 2 (*Vp2*), and the RGNNV coat protein (*Cp*) genes were used. qPCR reactions were prepared in a final volume of 25 μL, containing 1× SYBR Green Master Mix (Applied Biosystems, CA, USA), 10 μM primers, and 1 μL of cDNA template. All reactions were performed in technical triplicates on a 7300 Real-Time PCR System (Applied Biosystems, CA, USA) under standard qPCR cycling conditions. A melting curve analysis was included to confirm product specificity and detect viral replication. The *eukaryotic elongation factor 1 alpha* (*eef1a*) gene, which remains stable during infection [5], was used as the reference gene. Relative expression levels of target genes were normalized using the Pfaffl method [43]. Fold change values were calculated by dividing the normalized expression of each target gene in infected turbot samples by that of the corresponding control samples. Differences between infected and control fish were analyzed using the non-parametric Mann–Whitney U test in GraphPad Prism v. 5.02, with significance set at *p* < 0.05.

### 2.8. Isolation and Characterization of Head Kidney Leucocytes

Juvenile turbot (mean length: 12.6 ± 1.3 cm) were used to prepare primary cultures of total leukocytes. Briefly, head kidneys from three fish were aseptically removed and disaggregated by passing through a 40-μm nylon mesh in L-15 medium (Gibco, Thermo Fisher Scientific, Madrid, Spain) supplemented with penicillin/streptomycin (P/S), 2% fetal bovine serum (FBS), and heparin (10 U/mL; Gibco, Thermo Fisher Scientific, Madrid, Spain). The cell suspension was loaded onto a 51% Percoll density gradient (GE Healthcare, Uppsala, Sweden) and centrifuged at 500× *g* for 30 min at 4 °C. Cells at the interface were collected and washed in L-15 medium containing 0.1% FBS. Viability was assessed by staining with 0.1% trypan blue (Corning, NY, USA) and counting viable cells using a CellDrop FL cell counter (DeNovix Inc., Wilmington, DE, USA).

Leukocyte populations were characterized by flow cytometry and light microscopy. Flow cytometry analysis was performed on a FACS Calibur (BD Biosciences, San Jose, CA, USA) using the following acquisition settings: FSC at voltage E00, gain 5.25 (linear mode); SSC at voltage 350, gain 5.74 (log mode). One hundred thousand events were recorded per sample. Cell populations were identified in FSC/SSC density plots, sorted (R2 and R3 regions), and immobilized onto glass microscope slides by centrifugation in a Cytospin 4 (Epredia, Kalamazoo, MI, USA). Samples were fixed in 2% paraformaldehyde (PFA), stained with hematoxylin and eosin, and examined under a Nikon Eclipse 80i light microscope (Nikon Instruments Inc., Tokyo, Japan). The morphology of sorted cells included in the R2 and R3 regions was compared to that of unsorted whole-cell preparations.

Functional activities of leukocyte populations were assessed by evaluating phagocytic activity and ROS production by flow cytometry. Four cell cultures were prepared and seeded into 24-well plates at 1 × 10^6^ cells/mL. For phagocytosis assays, cells were incubated for 1 h at 18 °C with fluorescent latex beads (Thermo Fisher Scientific, Madrid, Spain) at a 1:1 ratio. Non-ingested particles were removed by two consecutive washes. Intracellular ROS production was evaluated by oxidation of the fluorescent probe CM-H_2_DCFDA (Thermo Fisher Scientific, Madrid, Spain) following stimulation with phorbol 12-myristate 13-acetate (PMA; 1 μg/mL; Sigma Aldrich, Madrid, Spain) for 1 h at 18 °C. In both assays, fluorescence was recorded in the FL-1 channel (530/30 nm) by flow cytometry.

### 2.9. Infection of Primary Cultures of Kidney Leucocytes

The effect of viral infections on ROS-related immune responses was evaluated in vitro. Primary cultures of total leukocytes were obtained from six animals and seeded at 1 × 10^6^ cells/mL into 24-well plates. Cell cultures were infected with VHSV, IPNV, or RGNNV at a multiplicity of infection (MOI) of 1. The production of total and mitochondrial ROS, as well as the release of NETs, was assessed as described below. Each experimental infection was performed twice.

### 2.10. Measurement of Total and Mitochondrial ROS Production

The production of total and mitochondrial ROS was assessed after both in vitro and in vivo viral infections. For in vitro assays, six primary cultures of total leukocytes were isolated and infected with VHSV, IPNV, or RGNNV at a MOI of 1, as described above. ROS production was measured at 24, 48, and 72 hpi. For in vivo assays, 20 fish were intramuscularly or intraperitoneally infected, as previously described and head kidneys were sampled at 3 dpi for ROS measurement in isolated leukocytes.

Total ROS production was determined by chemiluminescence in white 96-well plates (Thermo Fisher Scientific, Madrid, Spain), measuring relative luminescence units (RLU) after addition of 0.1 mM luminol (5-amino-2,3-dihydro-1,4-phthalazinedione; Sigma Aldrich, Madrid, Spain), alone or in combination with PMA (1 μg/μL; Sigma Aldrich, Madrid, Spain). Chemiluminescence was recorded for 1 h at 5 min intervals, with four technical replicates per sample, using a luminometer (GlowMax, Promega Biotech Iberica, Madrid, Spain). Differences between infected and control cells at each sampling point were analyzed by the non-parametric Kruskal–Wallis test followed by post hoc pairwise comparisons (GraphPad Prism v. 5.02).

Mitochondrial ROS (mtROS) production was quantified by flow cytometry after staining cells with 1 μM MitoSOX Green (Thermo Fisher Scientific, Madrid, Spain) for 30 min at room temperature. After two PBS washes, fluorescence was measured in a FACS Calibur (BD Biosciences, San Jose, CA, USA), with excitation at 488 nm and emission collected in the FL1 channel (530/30 nm, voltage 550). Forward scatter (FSC: voltage E00, gain 5.25, linear mode) and side scatter (SSC: voltage 287, gain 5.74, log mode) settings were used to gate viable cells, excluding debris. Results were expressed as shifts in mean fluorescence intensity (MFI) in histogram plots. Differences between the infected and control groups were analyzed using the nonparametric Mann–Whitney U test (GraphPad Prism v. 5.02). A *p*-value < 0.05 was considered significant.

### 2.11. Measurement of Neutrophil Extracellular Traps (NETs)

The release of NETs was evaluated in vivo and in vitro under the experimental conditions described above.

For the in vivo assay, a treatment with expression plasmids encoding two type I interferons (IFN I-1 and IFN I-2) was performed to generate a positive control and to validate NET response measurement. A total of 24 fish were used in this experiment. Expression plasmids were previously constructed [44]. Briefly, the nucleotide sequences encoding the mature IFN I-1 and IFN I-2 peptides were cloned into pMCV1.4 vectors, propagated in *E. coli* One Shot TOP10F′ competent cells (Invitrogen, Carlsbad, CA, USA), and purified using the PureLink™ HiPure Plasmid Midiprep Kit (Invitrogen, Carlsbad, CA, USA). Six juvenile turbots were intramuscularly injected with 2.5 μg (50 μL) of each plasmid, while control fish received the same volume of empty vector. At 48 hpi, head kidney leukocytes were isolated, seeded on poly-D-lysine (Sigma Aldrich, Madrid, Spain)–treated glass coverslips in 24-well plates (Falcon, BD Biosciences, San Jose, CA, USA) for 3 h, stimulated with PMA (2.5 μg/mL; Sigma Aldrich, Madrid, Spain) for 1 h, and fixed with 2% PFA (Sigma Aldrich, Madrid, Spain). Coverslips were then washed, stained with DAPI (1 μg/mL; Sigma Aldrich, Madrid, Spain), and mounted using ProLong Gold antifade reagent (Thermo Fisher Scientific, Madrid, Spain. Samples were examined by fluorescence microscopy using the Leica SPE (Leica Microsystems, Wetzlar, Germany), and the percentage of NETotic cells was determined from eight representative images based on chromatin decondensation.

NET release was also quantified by a fluorometric assay. Leukocytes (5 × 10^6^ cells/mL) from three fish were seeded in black 96-well plates (Thermo Fisher Scientific, Madrid, Spain) and stimulated with PMA (2.5 μg/mL; Sigma Aldrich, Madrid, Spain). After 3 h, NET-associated DNA was detected using 5 μM SYTOX Green (Thermo Fisher Scientific, Madrid, Spain) in a fluorometer (GlowMax, Promega Biotech Iberica, Madrid, Spain; excitation 485 nm, emission 538 nm) over 240 min, with measurements every 30 min. After the assay, cells were treated with 1% Triton X-100 (Roche Diagnostics, Mannheim, Germany) to determine the maximum DNA release and calculate the percentage of NET-associated DNA. Differences between the infected and control groups were analyzed using the nonparametric Mann–Whitney U test (GraphPad Prism v. 5.02). A *p*-value < 0.05 was considered significant.

### 2.12. Effect of ROS Modulation on Viral Replication

The involvement of ROS in viral replication was assessed using pharmacological inhibitors. Diphenyleneiodonium (DPI; Sigma Aldrich, Madrid, Spain), a general NADPH oxidase inhibitor, was used to block cytoplasmic ROS production, and the mitochondrial uncoupling agent 2,4-dinitrophenol (DNP; Sigma Aldrich, Madrid, Spain) was used to inhibit mitochondrial ROS production.

Non-toxic concentrations of the inhibitors were determined prior to infection experiments. Briefly, total leukocytes were seeded in 96-well plates and treated with DPI (50, 10, and 5 μM) or DNP (100, 50, and 25 μM). After 24 h of incubation, the modulators were removed and replaced with fresh medium. Cell viability was evaluated 24 h post-treatment using the MTT assay by adding 1 mM MTT (Sigma Aldrich, Madrid, Spain/Fisher) and measuring absorbance at 540 nm in a plate reader (GlowMax, Promega Biotech Iberica, Madrid, Spain) after 4 h incubation at 20 °C. The inhibitory effects of the selected concentrations were verified in five primary leukocyte cultures treated with DPI (10 and 5 μM) or DNP (100, 50, and 25 μM) for 24 h, and total ROS production was measured by chemiluminescence as previously described.

For viral replication assays, four leukocyte cultures were treated with non-toxic concentrations of DPI (10 and 5 μM) or DNP (100, 50, and 25 μM) for 24 h and then infected with VHSV, IPNV, or RGNNV at a MOI of 1, as previously described. Cells were collected at 24 and 48 hpi, and viral replication was quantified via qPCR. Differences in viral concentration between treated and control cells were analyzed using the non-parametric Mann–Whitney U test (GraphPad Prism v. 5.02). Statistical significance was considered at *p* < 0.05, *p* < 0.01, and *p* < 0.001.

## 3. Results

### 3.1. Protein Identification, Quantification, and Differential Abundance Analysis

**Figure 1:**
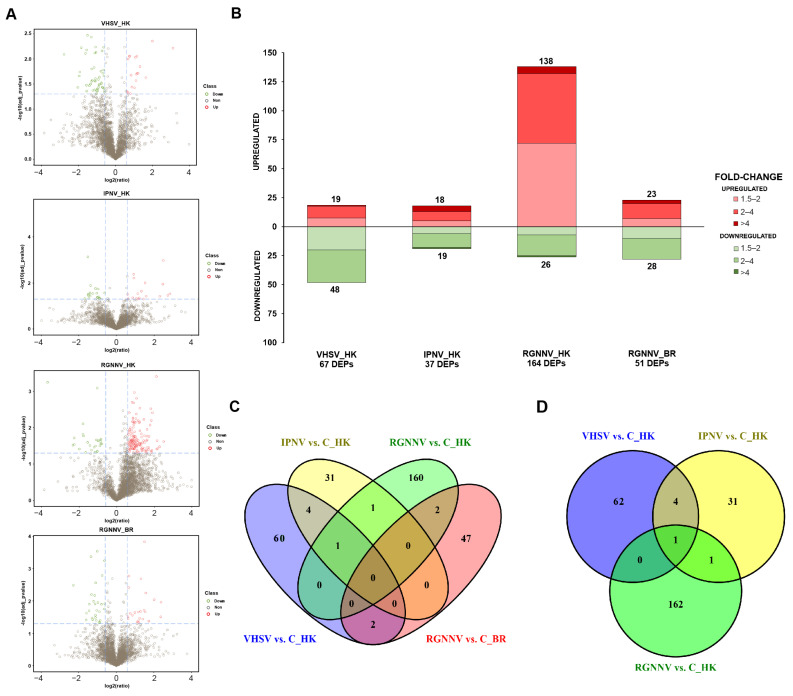
Differential protein abundance between virus-infected turbot and corresponding controls: (**A**) Volcano plots showing proteins with differential abundance. Green circles (left) and red circles (right) represent proteins with lower and higher abundance in infected animals, respectively, compared with controls. For each protein, significance (−log_10_[adjusted *p*-value]) is plotted against the log_2_(ratio) between groups. Dashed lines indicate the significance thresholds. The vertical axis represents the log2 fold change, and the horizontal axis corresponds to the *p*-value; (**B**) Stacked column charts illustrating the number of differentially expressed proteins (DEPs) and the magnitude of change (fold change); (**C**) Venn diagram showing shared and unique DEPs across comparisons; (**D**) Venn diagram excluding brain samples, showing shared and unique DEPs in kidney samples only.

Mass spectrometry analysis (MS) identified 44,704 peptides and 4499 proteins across all samples (Supplementary Table S2). Library characteristics, including the distribution of unique peptides, protein mass, and protein coverage, are shown in Supplementary Figure S1. Applying a fold change > 1.5 and adjusted *p* < 0.05, volcano plots and stacked column charts revealed 67 DEPs for VHSV vs. C_IP (19 up- and 48 downregulated), 37 for IPNV vs. C_IP (18 up- and 19 downregulated), 164 for RGNNV vs. C_IM kidney (138 up- and 26 downregulated), and 51 for RGNNV vs. C_IM brain (23 up- and 28 downregulated) (Figure 1A,B).

### 3.2. Proteins Affected by the Challenge with the Different RNA Viruses

Information on protein codes, annotations, fold changes, and adjusted *p*-values for the DEPs is available in Supplementary File S1. Venn diagram analyses revealed that no proteins were shared across all comparisons. When the brain was excluded, only one DEP, the neutrophil cytosol factor 4 (Ncf4), was common to the three viral challenges in the kidney (Figure 1C,D). The DEPs at 3 dpi varied by virus and, for RGNNV, also by tissue. The highest number of shared DEPs was found between the intraperitoneally inoculated viruses (VHSV and IPNV), which shared five DEPs: Ncf4, RNA-binding protein 4B (Rbm4b), splicing factor 3A subunit 3 (Sf3a3), glycerol-3-phosphate dehydrogenase mitochondrial (Gpd2), and endoplasmic reticulum aminopeptidase 1b (Erap1). Abundance patterns of DEPs were visualized using heatmaps, and protein–protein interaction networks were predicted with STRING. GO enrichment analyses were performed separately for each virus.

#### 3.2.1. VHSV

**Figure 2:**
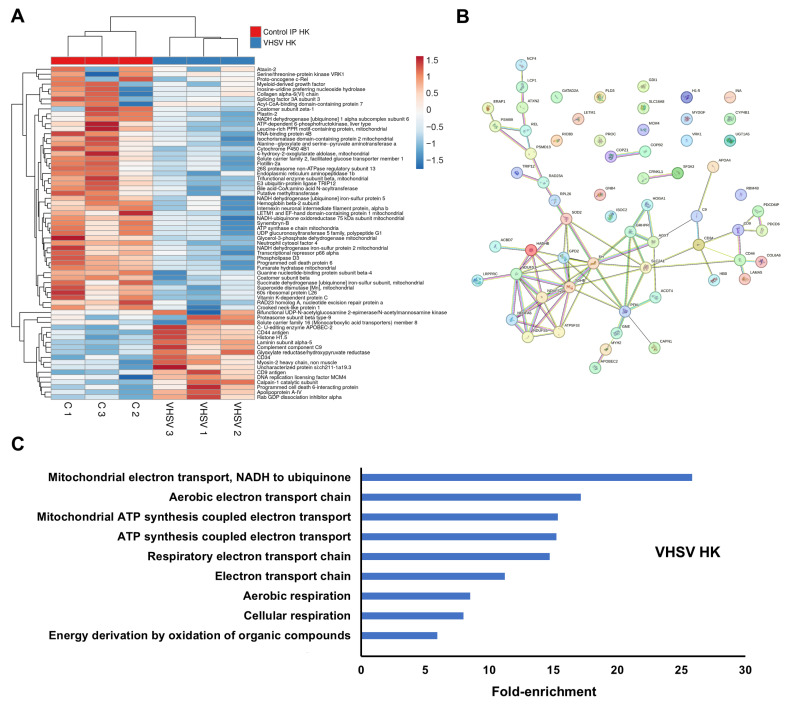
DEPs in the kidney at 3 dpi in VHSV-infected turbot compared with controls: (**A**) Heatmap showing DEP abundance following VHSV challenge; (**B**) Predicted protein–protein interaction network; (**C**) Gene Ontology enrichment analysis of significantly affected biological processes.

VHSV infection significantly altered the abundance of 67 proteins at three days post ip injection (Supplementary File S1). Only a small subset of immune-related proteins showed increased abundance relative to controls, including complement component C9, Cd34, Cd9, Cd44, and Apobec-2 (Figure 2A). Most DEPs, including immune proteins such as plastin-2 (Lcp1) and Ncf4, were downregulated (Figure 2A). The main protein–protein interaction cluster comprised mitochondrial electron transport chain (ETC) components: Complex I subunits (Ndufs1, Ndufs2, Ndufs5, Ndufa6), Complex II (Sdhb), Complex V (Atp5f1e), and proteins involved in ETC electron supply (Gpd2, Hadhb, Fh), regulation (Lrpprc), or detoxification (Sod2) (Figure 2B). GO enrichment analysis revealed nine significantly enriched biological process terms, all associated with ETC function (Figure 2C).

#### 3.2.2. IPNV

**Figure 3:**
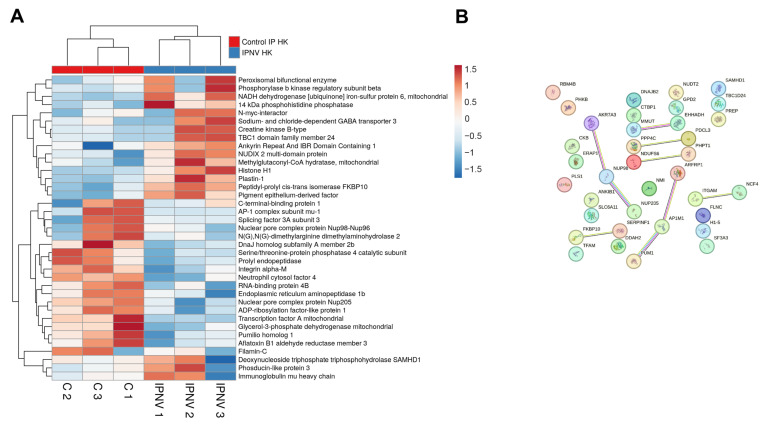
DEPs in the kidney at 3 dpi in IPNV-infected turbot compared with controls: (**A**) Heatmap showing DEP abundance following IPNV challenge; (**B**) Predicted protein–protein interaction network.

IPNV infection resulted in the differential expression of 37 proteins in the kidney at 3 dpi (Supplementary File S1). Upregulated immune-related proteins included Samhd1 and IghM (Figure 3A). Ncf4 was downregulated, consistent with the response observed during VHSV infection. No defined clusters were detected in the protein–protein interaction network (Figure 3B), and GO enrichment analysis revealed no significantly enriched biological process terms.

#### 3.2.3. RGNNV

**Figure 4:**
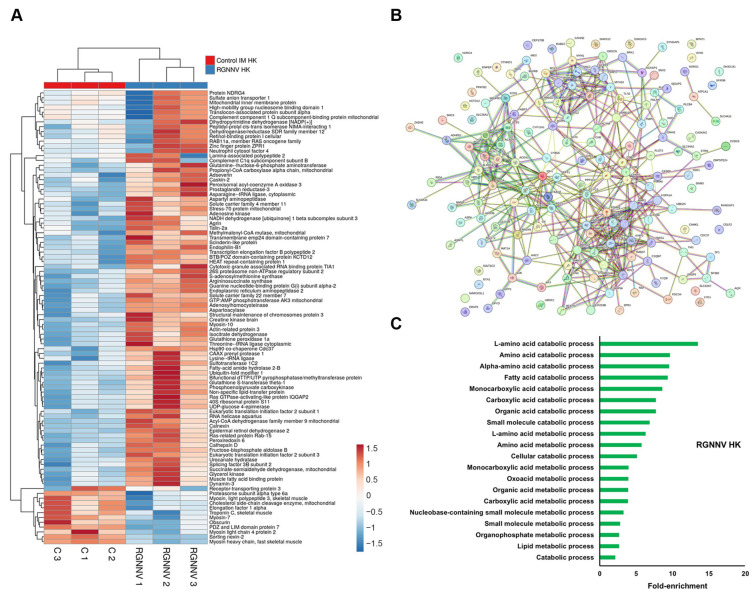
DEPs in the head kidney at 3 dpi in RGNNV-infected turbot compared with controls: (**A**) Heatmap showing DEP abundance following RGNNV challenge; (**B**) Predicted protein–protein interaction network; (**C**) GO enrichment analysis of significantly affected biological processes.

Although RGNNV is primarily neurotropic, its intramuscular injection induced the most extensive proteomic changes in kidney tissue among all viral challenges (Supplementary File S1). Immune-related proteins with increased abundance included two G-type lysozymes (Lyg1s), complement C1 subcomponents (C1qb and C1qbp), and membrane cofactor proteins (Mcp/Cd46 and Cd81) (Figure 4A). Notably, Ncf4 abundance was significantly increased relative to controls, in contrast to its downregulation during VHSV and IPNV infections. STRING network analysis identified three main clusters of interacting proteins (Figure 4B). The first cluster was enriched in cytoskeleton organization and muscle contraction proteins, including myosin family members (Myh1, Myh7, Myl2, Myl3, Myl4, Myo5a, Myh10), obscurin (Obscn), troponin C (Tnnc2), tropomodulin-2 (Tmod2), actin-related protein 3 (Actr3), actinin alpha 4 (Actn4), and filamin-B (Flnb), with both up- and downregulated members (Figure 4A). The second cluster comprised translation-related proteins (e.g., Rps11, Rpl5, Aimp2, Kars1, Tars1, Eif2s3b), which were generally upregulated post-infection (Figure 4B). The third cluster contained proteins involved in carboxylic acid metabolism (e.g., Ass1, Aldob, Pck1, Pcca, Idh1, Aldh5a1, Acad9, Adhfe1, Acox1, Acox3) and in antioxidant defense and ROS detoxification (e.g., Gpx1a, Prdx6, Mgst2, Gsstt1), all of which were upregulated (Figure 4A). GO enrichment analysis revealed multiple significantly enriched terms related to carboxylic acid metabolism, lipid metabolism, and amino acid catabolism (Figure 4C).

**Figure 5:**
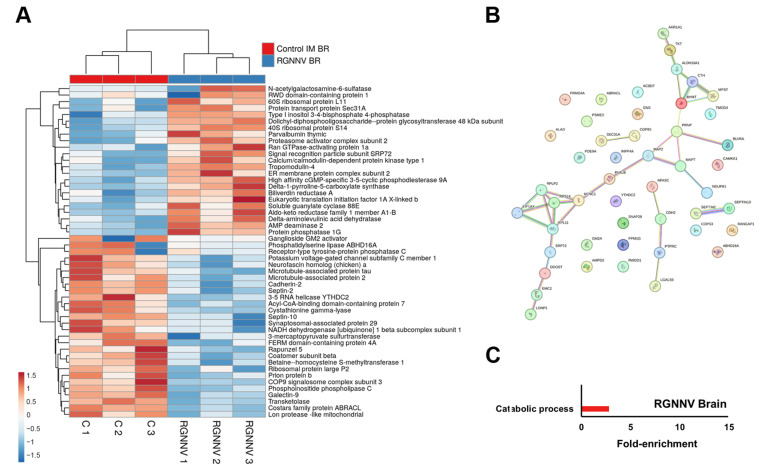
DEPs in the brain at 3 dpi in RGNNV-infected turbot compared with controls: (**A**) Heatmap showing DEP abundance following RGNNV challenge; (**B**) Predicted protein–protein interaction network; (**C**) GO enrichment analysis of significantly affected biological processes.

In the brain, immune-related DEPs included galectin-9 (Lgals9), receptor-type tyrosine-protein phosphatase C (Ptprc), cadherin-2, Septin-2, and Septin-10 (Supplementary File S1), all showing reduced abundance at 3 days following RGNNV infection (Figure 5A). With only 51 DEPs, the protein–protein interaction network showed no major clusters, although small groups were identified for protein synthesis (Rps14, Rpl11, Rplp2, Eif1ax) and amino acid and sulfur metabolism (Aldh18a1, Bhmt, Cth, Mpst) (Figure 5B). GO enrichment analysis identified a single significantly enriched term: “catabolic process” (Figure 5C).

### 3.3. Proteins Involved in ROS Production and Detoxification Are Affected by the Three RNA Viruses

Proteins involved in mitochondrial and cytoplasmic ROS production, as well as in ROS detoxification and redox balance, were affected by all three viruses (Table 1). During VHSV infection, several mitochondrial ETC components from Complex I, II, and V were reduced, as previously noted. The abundance of mitochondrial Sod2, responsible for superoxide detoxification, also decreased. In cytoplasmic ROS pathways, cytochrome P450 4B1 (Cyp4b1), involved in ROS generation during xenobiotic metabolism and fatty acid oxidation, and Ncf4, a regulatory subunit of the phagocyte NADPH oxidase complex, were downregulated (Table 1). In the kidney of RGNNV-infected turbot, proteins involved in mitochondrial and cytoplasmic ROS production and ROS detoxification were generally upregulated (Table 1). ETC-associated proteins Ndufb3 and Aldh5a1 showed increased abundance, as did cytoplasmic ROS-related proteins Cyb5, Acox1, Acox3, and Ncf4. Detoxification enzymes Gpx1a, Prdx6, Mgst2, Gstt1, Blvrb, and Adhfe1 were also elevated. In the brain, RGNNV increased the abundance of biliverdin reductase A (Blvra) but reduced the abundance of proteins linked to sulfur metabolism and oxidative stress regulation, including Ndufb1, Mpst, and Cth (Table 1). Finally, IPNV induced only mild changes in the abundance of ROS-related proteins (Table 1). Ndufs6 and Ehhadh were upregulated, whereas Gpd2, Ncf4, Ddah2, and Akr7a3 were downregulated in infected turbot (Table 1).

### 3.4. Temporal Expression Profiles of Genes Associated with Mitochondrial and Cytoplasmic ROS Production and Detoxification After Viral Challenge

**Figure 6:**
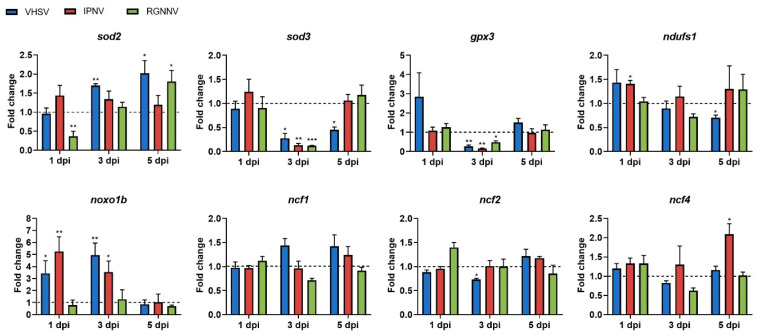
Modulation of genes involved in ROS detoxification and production in the kidney at 1, 3, and 5 days following infection with different RNA viruses. Expression levels were normalized to eef1a and expressed as fold change relative to controls (control-ip for VHSV/IPNV; control-im for RGNNV). Graphs show means ± SEM (*n* = 4 biological replicates). Significant differences: *** *p* < 0.001, ** *p* < 0.01, * *p* < 0.05.

The transcriptional response of redox-related genes revealed distinct, virus-specific patterns in the kidney (Figure 6). VHSV significantly upregulated *noxo1b* at early times post-infection (1 and 3 dpi), promoting cytoplasmic ROS generation, while suppressing key extracellular antioxidant genes *sod3* and *gpx3*, which may compromise redox buffering capacity. In mitochondria, a transient increase in *sod2* expression (3 dpi) was followed by inhibition of *ndufs1* (5 dpi), suggesting ETC impairment, potential electron leakage, and enhanced oxidative stress at later stages (Figure 6). IPNV increased *noxo1b* (1 and 3 dpi) and *ncf4* (5 dpi) expression but significantly reduced *sod3* and *gpx3* (3 dpi), without significant modulation of mitochondrial *sod2* or *ndufs1* (Figure 6). RGNNV caused no significant changes in cytoplasmic ROS-related genes (*noxo1b*, *ncf1*, *ncf2*, and *ncxf4*), but significantly downregulated *sod3* and *gpx3* (3 dpi), and transiently decreased *sod2* (1 dpi) before increasing its expression at 5 dpi (Figure 6).

### 3.5. Modulation of ROS Production in Both In Vitro and In Vivo Infection Models

**Figure 7:**
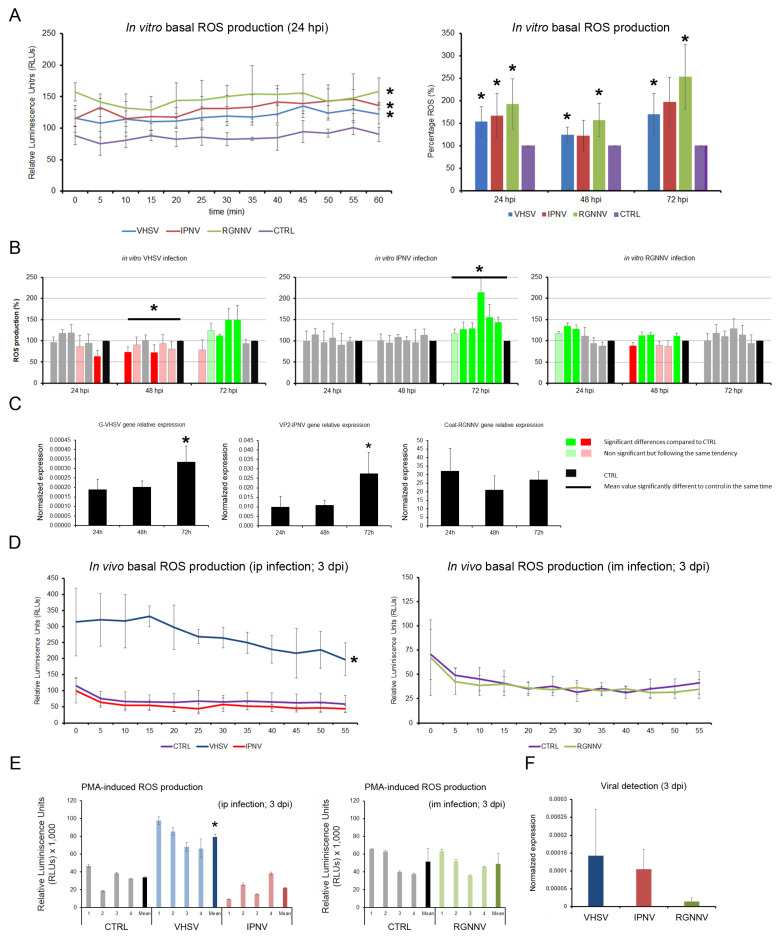
Total ROS production in kidney leukocytes at 24, 48, and 72 hpi: (**A**) Basal ROS levels measured by chemiluminescence, showing significant increases induced by all three viruses. (**B**) Modulation of PMA-induced ROS production following viral infection. Green and red bars indicate significant increases and decreases in ROS levels, respectively. (**C**) Relative viral gene expression measured by qPCR. (**D**) In vivo basal ROS production at 3 dpi. (**E**) Viral modulation of PMA-induced ROS production after in vivo infection. (**F**) Viral gene expression at 3 dpi. In vitro data are presented as means ± SD (*n* = 6); in vivo data as means ± SD (*n* = 4). Significant differences: * *p* < 0.05.

In vitro infection of total leukocytes with VHSV, IPNV, and RGNNV generally led to a significant increase in basal ROS production, except for IPNV at 48 and 72 hpi, where the changes were not significant, although they followed the same increasing trend (Figure 7A). PMA-induced ROS production was differentially modulated: VHSV significantly decreased it at 48 hpi and increased it at 72 hpi; IPNV increased it only at 72 hpi; RGNNV induced a weaker ROS response at the early stage of infection (24 hpi), returning to basal levels by 72 hpi (Figure 7B). The ROS peaks in VHSV- and IPNV-infected cells at 72 hpi coincided with maximal expression of the viral *G* and *Vp2* genes, respectively, whereas RGNNV-infected cells showed no significant changes in *Cp* gene expression (Figure 7C).

In vivo, only VHSV-infected fish displayed significantly elevated basal and PMA-stimulated ROS levels in head kidney leukocytes at 3 dpi (Figure 7D,E). IPNV induced a non-significant reduction in PMA-induced ROS, whereas RGNNV showed no effect. qPCR at 3 dpi confirmed the presence of VHSV and IPNV in leukocytes, with lower levels of RGNNV (Figure 7F).

### 3.6. Involvement of mtROS During Viral Infections

Mitochondrial ROS production was analyzed by flow cytometry. Three different cell populations were detected and characterized in total leukocytes isolated from the head kidney by flow cytometry (Supplementary Figure S2). The R3 region was composed of macrophages and large neutrophils with a diameter greater than 5 μm, as supported by the increased percentage of cells in this region when a specific Percoll gradient was used and confirmed by microscopy. Moreover, the highest phagocytic activity and subsequent intracellular ROS production were detected in this R3 region. The R2 region was mainly enriched in lymphocytes, although small numbers of neutrophils were also present. Finally, the R1 region consisted mainly of cell debris and small lymphocytes (Supplementary Figure S2).

**Figure 8:**
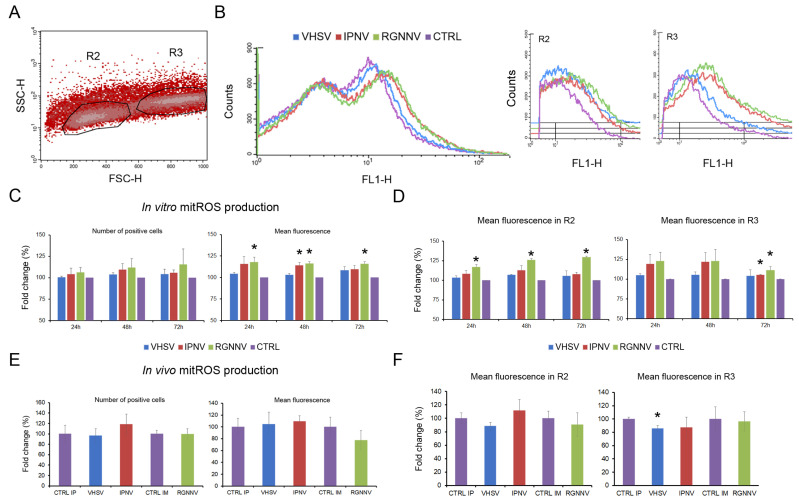
Mitochondrial ROS production in leukocytes at 24, 48, and 72 hpi: (**A**) MitoSOX Green fluorescence detected mainly in the R2 (lymphocytes and small neutrophils) and R3 (macrophages and large neutrophils) regions; (**B**) mtROS production visualized as shifts in mean fluorescence intensity histograms; (**C**) mtROS production after in vitro infection with three viruses, assessed as the percentage of positive cells and changes in mean fluorescence intensity; (**D**) In vitro mtROS production in R2 and R3 cell subsets; (**E**) In vivo mtROS production at 3 dpi, showing no significant changes in total percentage of positive cells or mean fluorescence intensity; (**F**) A significant reduction in mean fluorescence intensity in R3 cells after VHSV infection at 3 dpi. In vitro data are presented as means ± SD (*n* = 6); In vivo data as means ± SD (*n* = 4). Significant differences: * *p* < 0.05.

The production of mtROS was specifically measured using the MitoSOX Green staining. The fluorescent signal was detected primarily in both the R2 (lymphocytes and small neutrophils) and R3 (macrophages and large neutrophils) regions, indicating that all cell types produced mtROS at basal levels (Figure 8A). mtROS production was visualized as a shift in the mean fluorescence intensity, as shown in the histogram. This analysis was conducted using both the total fluorescence signal and the fluorescence emitted by the cells included in the R2 and R3 regions (Figure 8B).

In vitro infection of leukocytes modulated mtROS production. Although no differences in the percentage of fluorescent cells were observed throughout the experiment, infection with RGNNV at all time points and with IPNV at 72 hpi induced significant increases in mean fluorescence intensity (Figure 8C). The analysis of cell populations revealed that RGNNV significantly enhanced mtROS production in cells within the R2 region (lymphocytes and small neutrophils) at all time points, and also in macrophages and large neutrophils (R3 region) at the end of the experiment (72 hpi). Infection with IPNV induced significant changes only in macrophages and large neutrophils (R3 region) at 72 hpi. VHSV did not induce any significant change in mtROS production compared with control cells (Figure 8D). In vivo, no significant differences in total mtROS levels or in the percentage of fluorescent cells were detected at 3 dpi for any virus, except for a decrease in R3 macrophages and neutrophils from VHSV-infected fish (Figure 8E,F).

### 3.7. The Release of Neutrophil Extracellular Traps (NETs) Can Also Be Influenced by Viral Infections

**Figure 9:**
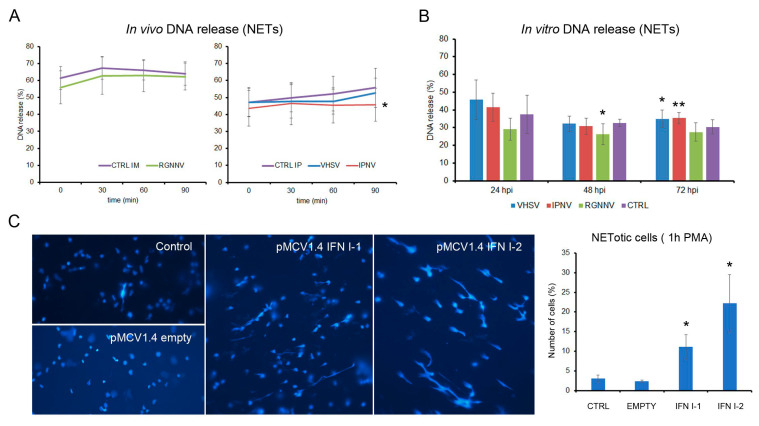
Induction of NET release following viral infection: (**A**) DNA release kinetics in leukocytes from fish infected in vivo at 3 dpi (*n* = 4); (**B**) DNA release after in vitro infection at 24, 48, and 72 hpi, measured 30 min after PMA stimulation (*n* = 3). (**C**) In vivo stimulation with plasmids expressing IFN-I1 or IFN-I2 significantly increased PMA-induced NET release from kidney leukocytes. Graph show mean ± SD from eight microscopic fields per sample. Significant differences: * *p* < 0.05, ** *p* < 0.01.

Chromatin decondensation and extracellular DNA release, which occur downstream of ROS production, were evaluated after viral infection and type I interferon (IFN-I) stimulation. Among the in vivo infections, only IPNV at 3 dpi significantly reduced PMA-induced DNA release, whereas VHSV and RGNNV had no effect (Figure 9A). In vitro, RGNNV decreased DNA release at 48 hpi, while VHSV and IPNV significantly enhanced it at 72 hpi (Figure 9B). In vivo, plasmid-driven expression of IFN-I1 or IFN-I2 significantly increased PMA-induced extracellular DNA release from kidney leukocytes (Figure 9C).

### 3.8. The Pharmaceutical Modulation of ROS Production Can Interfere with the Viral Replication

**Figure 10:**
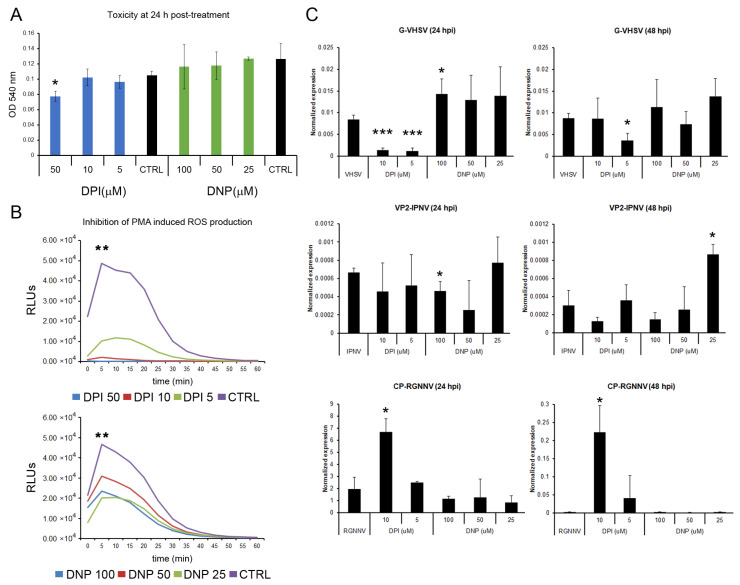
Effect of chemical inhibition of ROS production on viral replication: (**A**) DPI and DNP toxicity at 24 h was assessed by MTT assay in leukocytes. Graphs show mean ± SD (*n* = 2); (**B**) PMA-induced ROS production was significantly inhibited by DPI or DNP pretreatment. Representative data from two independent experiments using leucocytes from four fish are presented; (**C**) Viral replication was assessed by qPCR (viral gene expression). Graphs show mean ± SD (*n* = 4). Significant differences: *** *p* < 0.001, ** *p* < 0.01, * *p* < 0.05.

The role of ROS in viral replication was assayed in vitro by chemically blocking cytoplasmic and mitochondrial ROS production before infection. Non-toxic concentrations of DPI and DNP after 24 h of exposure were determined in leukocytes using the MTT assay. For DPI, only cells treated with 50 μM showed a significant reduction in cell viability. None of the DNP concentrations showed cytotoxic effects, and they did not affect cell viability (Figure 10A). The inhibition of ROS production was also verified. Treatment of leukocytes with DPI and DNP for 24 h significantly reduced PMA-induced ROS production. A dose-dependent inhibition was observed in cells treated with DPI, whereas no significant differences in ROS inhibition were detected among the different DNP concentrations (Figure 10B). The effect of the inhibitors was further evaluated by qPCR to analyze the viral genome replication (Figure 10C).

The replication of VHSV was affected by both inhibitors. At 24 hpi, treatment of cells with 10 and 5 μM DPI induced a significant reduction in *G* gene expression. In contrast, treatment of leukocytes with 100 μM DNP resulted in a significant increase in the *G* gene detection. DNP at 50 and 25 μM showed the same increasing trend. At 48 hpi, only treatment with 5 μM DPI significantly reduced *G* gene detection (Figure 10C). The replication of IPNV was also modulated by inhibitor treatment. Although treatment with DPI (10 and 5 μM) and DNP (100 and 50 μM) generally decreased *Vp2* gene detection, only 100 μM DNP caused a statistically significant reduction at 24 hpi. At the later time point (48 hpi), the highest concentrations of DPI and DNP (10 and 100 μM, respectively) also reduced *Vp2* gene expression, whereas 25 μM DNP significantly increased it (Figure 10C). The detection of the Cp gene of RGNNV was significantly increased in cells treated with 10 μM DPI at both 24 and 48 hpi. None of the other treatments significantly modify Cp gene expression (Figure 10C).

## 4. Discussion

The identification of common antiviral mechanisms by comparing immune responses is influenced by several factors, including host susceptibility, viral species, strain virulence, and environmental conditions [4,5,30,45,46,47]. Moreover, replication dynamics differ among viruses, leading to temporal variations in cellular responses that may not necessarily reflect virus-specific mechanisms [48,49]. To reduce variability, standardized infections in turbot with VHSV, IPNV, and RGNNV were performed using sublethal doses and comparable MOI values, and proteomic and functional analyses were combined to identify conserved immune responses and evaluate the role of redox balance during viral infection.

The quantitative analysis of the proteomic data revealed marked differences in the number of DEPs across infections; however, the magnitude of these changes did not correlate with the previously reported transcriptomic profiles from the same experiment [5]. This discrepancy was expected, as transcriptomics reflects mRNA abundance, whereas proteomics captures post-transcriptional and post-translational regulation that more closely reflects functional protein activity [50]. In addition, RNA-seq generally detects more modulated transcripts due to its broader genomic coverage and greater sensitivity compared with mass spectrometry [51]. Notably, RGNNV induced the strongest proteomic response in the head kidney, despite limited transcriptomic modulation, whereas VHSV produced the highest transcriptional changes, but only 67 DEPs, mostly downregulated. IPNV caused moderate effects on both omic levels. Interestingly, the brain, despite the neurotropic nature of RGNNV [52], displayed the fewest proteomic changes. This limited number of proteomic changes observed in the brain likely reflects the early stage of infection and the restricted proportion of infected neurons at these time points. More pronounced proteomic alterations may occur at later stages of infection, coinciding with extensive necrosis of neuronal tissue and a stronger inflammatory response. Overall, although transcriptomic responses to these viruses have been extensively described, proteomic remodeling and its implications for redox homeostasis within the same host species remain comparatively underexplored.

When DEPs from kidney samples were compared, only one protein was shared among the three viral infections, Ncf4 (p40phox), a cytosolic regulatory subunit of the NADPH oxidase complex. This common modulation suggests that ROS generation and redox homeostasis are key components of the host response to RNA virus infection, consistent with observations in several human viral models [53,54]. Interestingly, Ncf4 abundance increased during RGNNV infection but decreased during VHSV and IPNV infection, suggesting virus-specific regulation of NADPH oxidase-dependent ROS production or differences in infection kinetics at the sampling time point. Moreover, a detailed examination of ROS-related proteins revealed additional modulation of both ROS-generating and antioxidant pathways, underscoring the complex interplay between oxidative stress and antiviral defense mechanisms in fish.

At both the proteomic and transcriptomic levels, VHSV infection disrupted the cellular redox balance through multiple mechanisms. First, a broad suppression of the antioxidant capacity was evidenced by the downregulation of key extracellular antioxidant genes (*sod3* and *gpx3*) at 3 dpi, along with reduced Sod2 protein levels compared with controls. A similar inhibition of antioxidant defenses has been reported in the kidney of *Paralichthys olivaceus* infected with VHSV [55]. Second, the decreased abundance of ETC subunits in the proteome, together with the downregulation of the *ndufs1* gene, suggests mitochondrial dysfunction, ETC impairment, electron leakage, and enhanced oxidative stress at late stages of infection. Comparable mitochondrial alterations have been described for other rhabdoviruses, such as SVCV in EPC fish cells [56] and rabies virus in mammals [57], where mitochondrial dysfunction induces oxidative stress and suppress the mitochondrial antiviral signaling (MAVS) pathway [58]. Finally, components of the NADPH oxidase complex were also reduced by VHSV at 3 dpi, including Ncf4 and the gene *ncf2*. This, together with the lower abundance of Cyp4b1, indicates a potential decrease in cytoplasmic ROS production. However, as discussed below, VHSV also triggers ROS synthesis, suggesting that the magnitude and direction of ROS modulation may depend on viral virulence and infection dynamics, tipping the redox balance toward either viral advantage or host defense.

In contrast, RGNNV triggered a pronounced upregulation of proteins associated with both mitochondrial and cytoplasmic ROS generation, together with the activation of antioxidant and detoxification pathways in the head kidney. Interestingly, a recent proteomic study reported a decrease in antioxidant proteins in the serum of European sea bass (*Dicentrarchus labrax*) following vaccination with inactivated nodavirus [59]. Functional studies have further shown that RGNNV dynamically modulates ROS levels depending on the infection context: in vivo, ROS production in head kidney cells remained stable at 1 dpi but was markedly inhibited at 5 dpi, whereas in vitro infection induced a rapid ROS increase within 1 h, followed by suppression at later stages [60]. By contrast, infection of the grouper fin cell line (GF-1) with RGNNV significantly increased both the proportion of ROS-producing cells and intracellular peroxide levels at comparable time points [25,61]. Moreover, the use of ROS inhibitors or scavengers demonstrated that oxidative stress promotes RGNNV replication and progression [25,61]. In the brain, RGNNV elicited milder changes in ROS-related proteins, likely reflecting tight redox control to limit neuronal damage [62]. However, pronounced proteomic alterations may occur at later stages of infection. Nonetheless, this response appears host- and dose-dependent, as elevated ROS levels have been detected in the brains of highly susceptible Asian seabass (*Lates calcarifer*) during RGNNV infection [63].

IPNV infection induced only modest alterations in ROS-related proteins, although this virus has been previously associated with enhanced oxidative stress in host tissues through mitochondrial dysfunction, activation of NOX enzymes, and disruption of iron metabolism [64]. Moreover, during the mid to late stages of infection, IPNV has been shown to trigger ROS-mediated secondary necrosis and apoptosis, facilitating viral release [65]. Proteomic analysis revealed decreased levels of several key proteins involved in ROS generation and detoxification, including Gpd2, Ncf4, Ddah2, and Akr7a3, suggesting an overall downregulation of oxidative pathways in infected turbot. A diminished antioxidant capacity was also supported by transcriptomic results, which showed downregulation of major extracellular antioxidant genes such as *sod3* and *gpx3*.

Building on proteomic evidence indicating that ROS production and redox homeostasis constitute a conserved component of the host response to RNA virus infection in fish, and acknowledging that, in mammals, such responses can either enhance antiviral defenses or facilitate viral replication [52], we next investigated their functional relevance. Because proteomic data provide only a static view of the redox state at 3 dpi, we conducted complementary in vivo and in vitro time-course assays using total head kidney leukocytes to capture the temporal dynamics between ROS production and viral replication. The experimental design and sampling time points (up to 72 h) were intentionally selected to capture early events prior to the onset of extensive tissue damage, although longer experimental periods could reveal additional phases of ROS modulation. Flow cytometry results revealed that ROS production, phagocytosis, and NETs release arise from multiple immune cell types present in the head kidney, including macrophages and neutrophils.

In vitro, VHSV, IPNV, and RGNNV increased basal ROS production in infected leukocytes, indicating that these viruses modulate the oxidative status of host cells during infection, as reported for multiple viral systems [52,66,67]. The main differences in ROS production kinetics were observed when cells were stimulated with PMA prior to infection. VHSV triggered a biphasic response, consisting of an initial suppression of ROS production followed by a late induction at 72 hpi, similar to that described for other RNA viruses, such as respiratory syncytial virus (RSV) [68]. This pattern appears to be strain- and host-specific, as infection of turbot macrophages with the freshwater VHSV strain DK-3592B failed to induce RO production [69]. Biphasic modulation of the immune response has also been described for other cellular processes, such as the unfolded protein response during spring viremia of carp virus (SVCV) infection of the zebrafish cell line ZF4, where viruses avoid early immune activation to ensure efficient genome replication but later induce ROS production, promoting progeny release through ROS-mediated cell death [70].

ROS levels increased during IPNV infection exclusively at 72 hpi. This late ROS peak coincided with maximal viral gene expression, supporting a link between replication stage, viral load, and oxidative burst, as reported in RTG-2 cells infected with IPNV [71]. ROS production also appeared to be strain and host dependent, since infection of leukocytes isolated from rainbow trout kidney with IPNV Sp strain (serotype A1) did not induce ROS production [72]. In contrast, RGNNV infection induced only a transient, early increase in ROS production. Although this result might suggest a limited pro-oxidant activity of RGNNV, such a modest and short-lived response could be associated with the absence of detectable viral replication in leukocytes, as previously reported in sea bream (*Sparus aurata* L.) [73]. In this context, ROS production may be primarily triggered by extracellular Toll-like receptor (TLR) recognition of viral particles, at levels lower than those observed during active cytoplasmic viral replication, when mitochondrial destabilization typically enhances ROS generation [66].

In vivo infections did not produce marked increases in ROS production in fish infected with RGNNV or IPNV, suggesting a tightly regulated redox balance at the organismal level to prevent oxidative damage. In contrast, VHSV infection induced a pronounced elevation in both basal and PMA-stimulated ROS levels in kidney leukocytes, indicative of a strong activation of phagocyte oxidative mechanisms.

Mitochondria-derived ROS (mtROS) play important roles in physiological functions, including immune regulation [67]. Viruses can exploit mitochondria by disrupting their function, activating proinflammatory cascades, and suppressing MAVS to enhance replication [58,74]. During RGNNV infection in turbot, small lymphocytes exhibited sustained mtROS production throughout the infection, consistent with proteomic data showing an increased abundance of proteins essential for mtROS generation (Ndufb3, CytC, Aldh5a1, Fh). Mitochondrial involvement in RGNNV infection has been previously reported, including mtROS induction, loss of mitochondrial membrane potential, and necrotic cell death [75]. In IPNV infection, mtROS production in turbot was detected at 48 hpi, restricted to macrophages, and correlated with upregulation of Ndufs6 and downregulation of Gpd2, suggesting virus-driven remodeling of mitochondrial metabolism [76,77]. In contrast, VHSV did not induce mtROS in vitro, and in vivo infection led to reduced mitochondrial oxidative activity in macrophages, consistent with proteomic evidence of widespread mitochondrial dysfunction.

As part of conserved mechanisms, the canonical antiviral response includes, among its core elements, the synthesis of type I interferons (IFN-I) following the recognition of viral components, as well as the production of ROS during viral replication. Beyond their classical immunomodulatory effects, IFN-I pathways also promote oxidative responses, further increasing ROS levels [78]. Since the release of neutrophil extracellular traps (NETs) depends on ROS production [79], it is plausible that IFN-I in fish may act as an additional driver of NET formation during viral infection, as has been described in mammals [80].

NET release has been reported in turbot and other species following wounding, bacterial infection, or stimulation with molecules that mimic bacterial, viral, or fungal infections [81,82,83]. NET formation has also been observed during mammalian viral infections, where it contributes to inflammation and limits pathogen spread [84]. However, the role of this response during viral infections in fish has not yet been extensively studied.

In our experimental model, the release of extracellular DNA fibers was measured in mixed head kidney cell populations containing both neutrophils and macrophages. As this activity is a well-established function of neutrophils and macrophages [84], the observed response may reflect the activity of both cell types. The modulation of NET release following infection with VHSV, IPNV, and RGNNV was heterogeneous but temporally correlated with ROS production. In vitro, VHSV and IPNV triggered robust NET release at 72 hpi, consistent with significant activation of the oxidative extracellular response and the concomitant increase in ROS production at this time point. In contrast, RGNNV significantly reduced NET release at 48 hpi, when ROS levels had already decreased below those of the control group. Similarly, in vivo infection resulted in reduced DNA release only after IPNV infection at 3 dpi. The lack of a clear pattern of NET induction by the viruses, together with the opposite effects observed in vitro and in vivo in the case of IPNV, suggests that different viruses may employ diverse strategies to interfere with the interferon response.

When using expression plasmids, a clear correlation was observed between type I IFN antiviral activity, ROS induction, and NET release. In vivo stimulation of turbot with two different type I IFNs strongly enhanced NET release, highlighting the role of IFN-I in priming leukocytes for oxidative activation. A higher number of NETotic cells was detected in fish stimulated with IFN-I2 compared to IFN-I1. This observation is consistent with Pereiro et al. [44], who reported that IFN-I2 participates in regulating inflammatory processes and enhances interleukin-1 beta expression. IL-1β can trigger ROS production by activating enzymes such as NADPH oxidase, while ROS, in turn, stimulate the inflammasome, thereby amplifying IL-1β release [85,86].

Pharmacological modulation of oxidative pathways, by inhibiting cytoplasmic or mitochondrial ROS production, influences viral replication in a virus-specific manner [52]. In our model, inhibition of cytoplasmic ROS by DPI treatment impaired the replication of VHSV and IPNV, supporting the dependency of these viruses on cytoplasmic ROS for efficient viral transcription, as previously reported using other antioxidant compounds such as curcumin [87] and α-lipoic acid [88]. Unexpectedly, cytoplasmic ROS inhibition enhanced the expression of the *Cp* gene in RGNNV-infected cells at both early and late stages, suggesting that basal ROS may normally constrain RGNNV replication. This finding contradicts previous reports showing that the use of ROS inhibitors such as DPI and N-acetylcysteine (NAC), or the overexpression of antioxidant enzymes, significantly reduced RGNNV viral titers in GF-1 cells [25,61]. Indeed, the infection model or kinetics could significantly influence these outcomes; therefore, results should be interpreted with caution before drawing definitive conclusions. Conversely, the inhibition of mitochondrial ROS with DNP enhanced VHSV *G* and IPNV *Vp2* gene expression, suggesting that maintaining mitochondrial integrity and functionality is essential for viral replication during the early stages of infection [66].

## 5. Conclusions

Our combined proteomic and functional analyses offer new insights into the role of ROS and redox homeostasis during RNA virus infections in turbot. Despite their distinct pathologies, VHSV, IPNV, and RGNNV all modulate redox balance, reinforcing the idea that oxidative regulation represents a conserved and central node in viral pathogenesis and/or antiviral responses in fish. Understanding how ROS contributes to both viral replication and host defense may inform the development of novel therapeutic strategies. Indeed, our results indicate that experimental manipulation of ROS levels can influence viral replication, suggesting a potential avenue for antiviral intervention; however, the risks associated with altering such a central process involved in multiple cellular functions must be carefully evaluated. Collectively, these findings emphasize that ROS production and redox regulation constitute a conserved yet finely tuned component in viral infections, where the magnitude, timing, and subcellular source of ROS critically shape infection outcomes. This study establishes a framework for future investigations into the interplay between oxidative signaling and viral pathogenesis in aquaculture species.

## Figures and Tables

**Table 1:** Proteins involved in mitochondrial or cytoplasmic ROS production, as well as in ROS detoxification and cellular redox balance, are modulated by different RNA viruses at 3 dpi. Red shading indicates a decrease in protein abundance after infection, whereas green shading indicates an increase following the viral challenge. HK: head kidney. BR: brain.

	VHSV HK	IPNV HK	RGNNV HK	RGNNV BR
**Mitochondrial ROS production**				
NADH-ubiquinone oxidoreductase 75 kDa subunit, mitochondrial (Ndufs1)	**DOWN**			
NADH dehydrogenase iron-sulfur protein 2, mitochondrial (Ndufs2)	**DOWN**			
NADH dehydrogenase [ubiquinone] iron-sulfur protein 5 (Ndufs5)	**DOWN**			
NADH dehydrogenase [ubiquinone] iron-sulfur protein 6, mitochondrial (Ndufs6)		**UP**		
NADH dehydrogenase [ubiquinone] 1 alpha subcomplex subunit 6 (Ndufa6)	**DOWN**			
NADH dehydrogenase [ubiquinone] 1 beta subcomplex subunit 1 (Ndufb1)				**DOWN**
NADH dehydrogenase [ubiquinone] 1 beta subcomplex subunit 3 (Ndufb3)			**UP**	
Cytochrome c (Cycs)			**UP**	
Succinate dehydrogenase [ubiquinone] iron-sulfur subunit, mitochondria (Sdhb)	**DOWN**			
Succinate-semialdehyde dehydrogenase, mitochondrial (Aldh5a1)			**UP**	
Fumarate hydratase, mitochondrial (Fh)	**DOWN**		**UP**	
Isochorismatase domain-containing protein 2, mitochondrial (Isoc2)	**DOWN**			
Glycerol-3-phosphate dehydrogenase, mitochondrial (Gpd2)	**DOWN**	**DOWN**		
**Cytoplasmic ROS production**				
Cytochrome b5 (Cyb5)			**UP**	
Peroxisomal acyl-coenzyme A oxidase 1 (Acox1)			**UP**	
Peroxisomal acyl-coenzyme A oxidase 3 (Acox3)			**UP**	
Neutrophil cytosol factor 4 (Ncf4)	**DOWN**	**DOWN**	**UP**	
Cytochrome P450 4B1 (Cyp4b1)	**DOWN**			
**ROS detoxification and cellular redox balance**				
Superoxide dismutase [Mn], mitochondrial (Sod2)	**DOWN**			
Glutathione peroxidase 1a (Gpx1a)			**UP**	
Peroxiredoxin 6 (Prdx6)			**UP**	
Microsomal glutathione S-transferase 2 (Mgst2)			**UP**	
Glutathione S-transferase theta-1 (Gstt1)			**UP**	
Hydroxyacid-oxoacid transhydrogenase, mitochondrial (Adhfe1)			**UP**	
3-Mercaptopyruvate sulfurtransferase (Mpst)				**DOWN**
Biliverdin reductase B (Blvrb)			**UP**	
Biliverdin reductase A (Blvra)				**UP**
N(G), N(G)-dimethylarginine dimethylaminohydrolase 2 (Ddah2)		**DOWN**		
Aflatoxin B1 aldehyde reductase member 3 (Akr7a3)		**DOWN**		
Peroxisomal bifunctional enzyme (Ehhadh)		**UP**		
Cystathionine gamma-lyase (Cth)			**UP**	**DOWN**

## Data Availability

The mass spectrometry proteomics data have been deposited to the Proteo-meXchange Consortium via the PRIDE [37] partner repository with the dataset identifier PXD06803.

## Supplementary Materials

The following supporting information can be downloaded at: https://www.mdpi.com/article/10.3390/antiox15010096/s1, File S1: Information on protein codes, annotations, fold changes, and adjusted *p*-values for the DEPs; Table S1: Sequences of primers used for gene expression analysis and viral replication detection; Table S2: Summary of sample-specific proteomic identifications, including sample name, total spectra acquired, identified spectra, number of unique peptides, and total proteins identified; Figure S1: Overview of library identification metrics. The figure presents key distributions obtained from the proteomic library analysis; Figure S2: Characterization of cells isolated from the head kidney.

## Abbreviations

The following abbreviations are used in this manuscript: 40S ribosomal protein S14 (Rps14); 60S ribosomal protein L11 (Rpl11); 60S ribosomal protein L5 (Rpl5); 3-mercaptopyruvate sulfurtransferase (Mpst); Acyl-CoA dehydrogenase family member 9 mitochondrial (Acad9); Aflatoxin B1 aldehyde reductase member 3 (Akr7a3); Aldehyde dehydrogenase 18 family member A1 (Aldh18a1); Aldehyde dehydrogenase 5 family member A1 mitochondrial (Aldh5a1); Aminoacyl tRNA synthase complex-interacting multifunctional protein 2 (Aimp2); Argininosuccinate synthase (Ass1); ATP synthase F1 subunit epsilon (Atp5f1e); Betaine-homocysteine S-methyltransferase 1 (Bhmt); Biliverdin reductase B (Blvrb); Complement C1 subcomponent subunit B (C1qb); Complement component 1 Q subcomponent-binding protein, mitochondrial (C1qbp); Cytochrome b5 (Cyb5); Cystathionine gamma-lyase (Cth); Deoxynucleoside triphosphate triphosphohydrolase (Samhd1); Dimethylarginine dimethylaminohydrolase 2 (Ddah2); Eukaryotic translation initiation factor 1A X-linked b (Eif1ax); Eukaryotic translation initiation factor 2 subunit 3b (Eif2s3b); Fructose-bisphosphate aldolase B (Aldob); Fumarate hydratase mitochondrial (Fh); Glutathione peroxidase 1a (Gpx1a); Glutathione S-transferase theta-1 (Gstt1); Glycerol-3-phosphate dehydrogenase mitochondrial (Gpd2); G-type lysozyme (Lyg1); Hydroxyacid-oxoacid transhydrogenase mitochondrial (Adhfe1); Immunoglobulin mu heavy chain (IghM); Isocitrate dehydrogenase (Idh1); Leucine-rich PPR motif-containing protein, mitochondrial (Lrpprc); Lysine-tRNA ligase (Kars1); Membrane cofactor protein (Mcp/Cd46) and Cd81; Microsomal glutathione S-transferase 2 (Mgst2); NADH dehydrogenase [ubiquinone] 1 alpha subcomplexes (Ndufs); NADH dehydrogenase [ubiquinone] 1 beta subcomplex subunit 1 (Ndufb1); NADH dehydrogenase [ubiquinone] 1 beta subcomplex subunit 3 (Ndufb3); NADH dehydrogenase [ubiquinone] iron-sulfur protein 6 mitochondrial (Ndufs6); Neutrophil cytosol factor 4 (Ncf4); Peroxiredoxin 6 (Prdx6); Peroxisomal acyl-coenzyme A oxidase (Acox) and 3 (Acox3); Peroxisomal bifunctional enzyme (Ehhadh); Phosphoenolpyruvate carboxykinase (Pck1); Plastin-2 (Lcp1); Propionyl-CoA carboxylase alpha chain mitochondrial (Pcca); Ribosomal protein large P2 (Rplp); Ribosomal protein S11 (Rps11); Succinate dehydrogenase [ubiquinone] iron-sulfur subunit, mitochondrial (Sdhb); Superoxide dismutase [Mn], mitochondrial (Sod2); Threonine-tRNA ligase 1 cytoplasmic (Tars1); Trifunctional enzyme subunit beta mitochondrial (Hadhb).
