# Supplementary material for: The Regulation of Oxidative Stress Is a Conserved Response to RNA Virus Infection in Fish
Source: Antioxidants (Basel). 2026 Jan 12;15(1):96. doi: 10.3390/antiox15010096 (PMC12837395; doi:10.3390/antiox15010096)
Supplement: Supplementary file 1 [file antioxidants-15-00096-s001.zip › supplementary tables.pdf]

antioxidants

MDPI

Supplementary data

Supplementary Table S1. Sequences of primers used for gene expression analysis and viral replication detection. Gene accession numbers and slope values for primer efficiency calculation have also been included.

Gene name<br>Gene accession number	Symbol<br>Slope	Forward	Reverse
Superoxide dismutase 2<br>ENSSMAG00000017117	*sod2*<br>-3.39	GGCTGGGCTATGACAAGGAG	TCTGCCGTGTTTCCTCTGTC
Superoxide dismutase 3<br>ENSSMAG00000008541	*sod3*<br>-3.551	GGCAGGCAGTCAACAATGTG	AGTGAGGTGATGGGTCGGAT
Glutathione peroxidase 3<br>ENSSMAG00000008452	*gpx3*<br>-3.552	CTCGGACTCACTGTTCTCGG	AATCCGTTGCCTGGTCTGAC
NADH-ubiquinone oxidoreductase<br>ENSSMAG00000015811	*ndufs1*<br>-3.6306	GACTGGCAGAAGTTTCCCCA	CAGCTTTCACACACTTGGCC
NADPH oxidase organizer 1b<br>ENSSMAG00000009040	*noxo1b*<br>-3.7148	GGTGGAAGTGGATGAGAGGC	CTCCTCACGGCACAGTACAG
Neutrophil cytosolic factor 1<br>ENSSMAG00000010703	*ncf1*<br>-3.4143	TCATCACCACCAACGCCTAC	CTCCTTTCCTGACGACCCAC
Neutrophil cytosolic factor 2<br>ENSSMAG00000001354	*ncf2*<br>-3.3209	ACCCCACACTGTCCTGTTTG	CCAGTTGTCAGCACCCTTCT
Neutrophil cytosolic factor 4<br>ENSSMAG00000021193	*ncf4*<br>-3.577	CCACCTACCCGCAAAGTGAA	TCTTCAGGTTCAACTCCGCC
Eukaryotic elongation factor 1ª<br>XM_035620146.2	*eef1a*<br>-3.32	GGAGGCCAGCTCAAAGATGG	ACAGTTCCAATACCGCCGATTT
VHSV glycoprotein gene<br>AY546628.1	G-VHSV<br>-3.4	CCCCTCGGATTGGTCATTC	AACAACAGCAAAGTACCAATAGAAGGA
IPNV viral polypeptide 2 gene<br>MH614930.1	*Vp2*-IPNV<br>-3.4	GCCAAGATGACCCAGTCCAT	TGACAGCTTGACCCTGGTGAT
RGNNV capsid protein gene<br>JN662462.1	*Cp*-RGNNV<br>-3.4	GACGCGCTTCAAGCAACTC	CGAACACTCCAGCGACACAGCA

Supplementary Table S2. Summary of sample-specific proteomic identifications, including sample name, total spectra acquired, identified spectra, number of unique peptides, and total proteins identified.

Sample	Total spectra	Identified spectra	Identified peptides	Identified proteins
**Control_IP_1**	84851	32102	20619	3529
**Control_IP_2**	82379	30383	19049	3310
**Control_IP_3**	81896	29377	20215	3547
**VHSV_1**	83910	30233	18981	3367
**VHSV_2**	79056	25575	17354	3240
**VHSV_3**	82820	28093	18213	3295
**IPNV_1**	82116	27443	17587	3223
**IPNV_2**	81663	26627	18019	3356
**IPNV_3**	82721	27556	18594	3351
**Control_IM_HK_1**	84315	31539	20142	3480
**Control_IM_HK_2**	84359	31985	20125	3491
**Control_IM_HK_3**	85360	31625	19144	3407
**RGNNV_HK_1**	82239	10015	9319	2943
**RGNNV_HK_2**	84483	30640	19485	3583
**RGNNV_HK_3**	85696	32798	19717	3543
**Control_IM_BR_1**	85838	32728	19522	3668
**Control_IM_BR_2**	85515	31373	19218	3631
**Control_IM_BR_3**	85256	31796	19620	3643
**RGNNV_BR_1**	85613	31355	18893	3620
**RGNNV_BR_2**	85533	31726	19489	3649
**RGNNV_BR_3**	84865	29736	19089	3583

https://doi.org/10.3390/antiox15010096
