# Supplementary material for: The Regulation of Oxidative Stress Is a Conserved Response to RNA Virus Infection in Fish
Source: Antioxidants (Basel). 2026 Jan 12;15(1):96. doi: 10.3390/antiox15010096 (PMC12837395; doi:10.3390/antiox15010096)
Supplement: Supplementary file 1 [file antioxidants-15-00096-s001.zip › supplementary figures.pdf]

antioxidants

MDPI

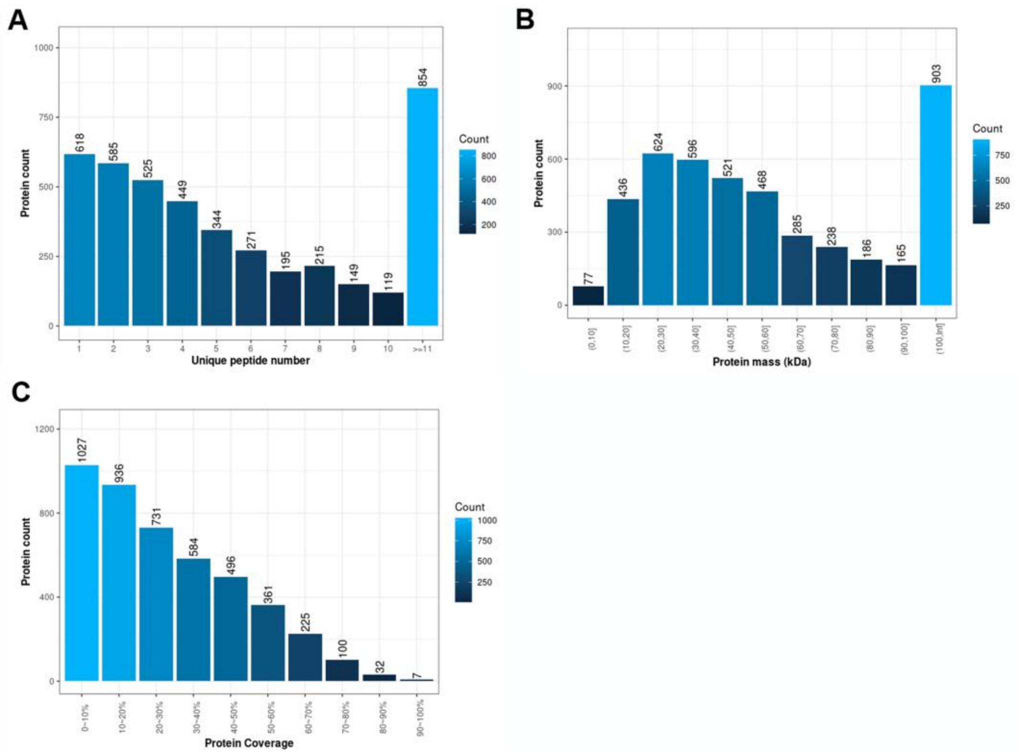

Supplementary Figure S1. Overview of library identification metrics. The figure presents key distributions obtained from the proteomic library analysis: (**a**) Distribution of unique peptides across the dataset, highlighting the diversity of peptide identifications; (**b**) Protein mass distribution, showing the range and prevalence of proteins identified according to their molecular weight; (**c**) Protein coverage distribution, representing the percentage of sequence coverage achieved for the identified proteins.

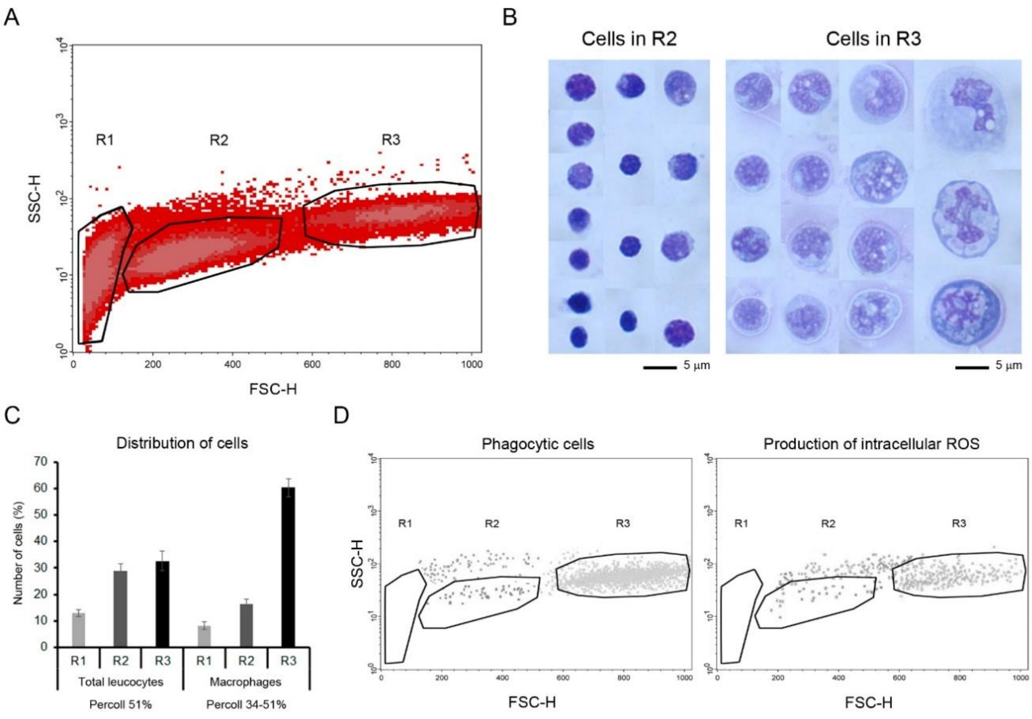

Supplementary Figure S2. Characterization of cells isolated from the head kidney: (**a**) Three distinct cell populations were identified in head kidney samples using a Percoll gradient; (**b**) The R1 region consisted mainly of cell debris and small lymphocytes, while the R2 region was predominantly enriched in lymphocytes, with a small number of neutrophils also present. The R3 region contained macrophages and neutrophils larger than 5 µm in diameter; (**c**) Total leukocytes was obtained from four head kidneys using a 51% Percoll gradient, and enrichment in macrophages and neutrophils was achieved using a 34-51% gradient; (**d**) Functional assays showed that the higher phagocytic activity (fluorescent latex bead uptake) and ROS production occurred in the R3 region, supporting the predominance of macrophages and neutrophils in this population.

https://doi.org/10.3390/antiox15010096
